# Innovative research methodologies in the EU regulatory framework: an analysis of EMA qualification procedures from a pediatric perspective

**DOI:** 10.3389/fmed.2024.1369547

**Published:** 2024-03-28

**Authors:** Viviana Giannuzzi, Arianna Bertolani, Silvia Torretta, Giorgio Reggiardo, Eleonora Toich, Donato Bonifazi, Adriana Ceci

**Affiliations:** ^1^Department of Research, Fondazione per la Ricerca Farmacologica Gianni Benzi Onlus, Bari, Italy; ^2^Department of Project Development, Consorzio per Valutazioni Biologiche e Farmacologiche (CVBF), Pavia, Italy; ^3^TEDDY, European Network of Excellence for Paediatric Research, Pavia, Italy

**Keywords:** innovative methodologies, pediatric research, regulatory, qualification procedure, European Medicines Agency

## Abstract

**Introduction:**

The European Medicines Agency (EMA) offers scientific advice to support the qualification procedure of novel methodologies, such as preclinical and *in vitro* models, biomarkers, and pharmacometric methods, thereby endorsing their acceptability in medicine research and development (R&D). This aspect is particularly relevant to overcome the scarcity of data and the lack of validated endpoints and biomarkers in research fields characterized by small samples, such as pediatrics.

**Aim:**

This study aimed to analyze the potential pediatric interest in methodologies qualified as “novel methodologies for medicine development” by the EMA.

**Methods:**

The positive qualification opinions of novel methodologies for medicine development published on the EMA website between 2008 and 2023 were identified. Multi-level analyses were conducted to investigate data with a hierarchical structure and the effects of cluster-level variables and cluster-level variances and to evaluate their potential pediatric interest, defined as the possibility of using the novel methodology in pediatric R&D and the availability of pediatric data. The duration of the procedure, the type of methodology, the specific disease or disease area addressed, the type of applicant, and the availability of pediatric data at the time of the opinion release were also investigated.

**Results:**

Most of the 27 qualifications for novel methodologies issued by the EMA (70%) were potentially of interest to pediatric patients, but only six of them reported pediatric data. The overall duration of qualification procedures with pediatric interest was longer than that of procedures without any pediatric interest (median time: 7 months vs. 3.5 months, respectively; *p* = 0.082). In parallel, qualification procedures that included pediatric data lasted for a longer period (median time: 8 months vs. 6 months, respectively; *p* = 0.150). Nephrology and neurology represented the main disease areas (21% and 16%, respectively), while endpoints, biomarkers, and registries represented the main types of innovative methodologies (32%, 26%, and 16%, respectively).

**Discussion:**

Our results underscore the importance of implementing innovative methodologies in regulatory-compliant pediatric research activities. Pediatric-dedicated research infrastructures providing regulatory support and strategic advice during research activities could be crucial to the design of *ad hoc* pediatric methodologies or to extend and validate them for pediatrics.

## Introduction

Innovative methodologies, including pharmacometrics, innovative trial designs, personalized medicine, biomarkers, preclinical models, and *in vitro* models, provide effective and valuable avenues for generating robust research evidence in today's context. The leveraging real-world data and registries has also been recognized as an “innovative way” to an innovative approach to generate evidence for scientific health research, to complement or even replace the traditional clinical research setting (1).

Over the past decades, researchers and companies have increasingly proposed new research methodologies to gain evidence in the biomedical field. These methodologies have the potential to reduce the time and efforts required to identify the failure of successful drugs early (2–8).

For this reason, regulators encourage the implementation of new methods for conducting research and development (R&D) programs (9–15). To facilitate this, voluntary regulatory procedures have been established to endorse the acceptability of a novel methodology not yet integrated into medicines R&D and clinical management, including the Food and Drug Administration (FDA), qualification of Drug Development Tools (DDT), and the European Medicines Agency (EMA) qualification procedure of novel methodologies for medicine development (16–18). The EMA qualification procedure is in charge of changing under the remit of the EMA Committee for Medicinal Products for Human Use (CHMP) and/or the EMA Scientific Advice Working Party (SAWP). The procedure leads to a qualification opinion (QO) or a qualification advice (QA), based on the assessment of the submitted data. The former establishes the acceptability of a specific use of the method under evaluation (e.g., use of a novel methodology or a novel biomarker); the latter is adopted when the data submitted for qualification are still preliminary and not sufficiently supportive, but promising. In this case, further investigations and data sharing are encouraged by providing a letter of support. Notably, prior to the final QO decision, the procedure is opened to the public consultation of the scientific community, aiming to expand scientific scrutiny and discussion. All the steps of the qualification procedure take a maximum of 190 days (19). According to the EMA annual report, 21 qualification requests for novel technologies have been submitted in 2022, with a rising trend from 2018 (20). Since 2005, the EMA and FDA have accepted joint applications for qualifications for biomarkers and clinical outcome assessments, aiming to improve the harmonization of international guidelines.^1^ Innovative research methodologies represent an opportunity to address the well-known challenges in the field of research and scientific progress characterized by small samples, especially in pediatrics and rare diseases. These challenges include the lack of science, scarcity of data, unavailability of proper preclinical models, age-related differences in pediatrics, lack of validated endpoints and biomarkers, geographic dispersion of experts, and specialized centers dealing with specific conditions (21).

For example, pharmacometrics methods, such as physiologically based pharmacokinetic (PBPK) modeling, are today increasingly utilized to help in defining doses for pediatric patients (22–26) and in first-in-human trials and to predict interactions between medicines (27–30). Moreover, pharmacometrics methods are expected to play a crucial role in other aspects of the medicine R&D, such as benefit–risk analysis (31, 32), to address the choice of a target molecule, optimize pre-clinical and clinical planning, and guide decision-making for future studies (3). This emphasizes the need for standardized approaches in pharmacometrics to enhance the quality and reproducibility of research in this field (11, 33), as well as the need for training to develop a skilled workforce in pharmacometrics (34).

Innovative trial designs are invented and tested as an alternative to the “golden standard” randomized controlled trial (RCT), aiming to identify responders with a small sample size while maintaining adequate statistical power. Starting from the EMA guidelines on clinical trials in small populations (35), master protocols (umbrella, platform, and basket trials) (8), cross-over and adaptive designs, sequential designs, n-of-1 trials, and randomized withdrawn designs can generate evidence to support the assessment of medicines (6, 36).

Personalized medicine approaches have the potential to effectively address the issue of diseases affecting small populations, so they can better find effective and reliable treatments and improve diagnostic outcomes in this field (37).

Other innovative tools and methods have been deemed useful to conduct pediatric studies (38).

In this work, we aimed to analyze the pediatric interest in methodologies qualified as “novel methodologies for medicine development” by the EMA since the introduction of this procedure. We also examined the duration of the procedure, the type of methodology, the disease or disease area addressed, and the type of applicant of the qualification opinions, with and without pediatric interest. Finally, we assessed the availability of pediatric data at the time of the opinion release.

## Materials and methods

### Sample

For this study, all the positive qualification opinions of novel methodologies for medicine development released between 1 January 2008 (i.e., since the implementation of the regulatory procedure in the EU) and 31 December 2023 were sourced from the EMA website^2^ and included in this study. Procedures with a draft QO and without the date of the final adoption by CHMP were not considered.

### Data extraction

The opinion letters for all the procedures were consulted on the EMA website and analyzed to extract the following data:

– the type of applicant;
– the type of methodology;
– any specific medicinal product, disease, or disease area addressed;
– the availability of pediatric data at the time of the opinion release; and
– the duration of the procedure.

### Data characterization and interpretation

The potential ***pediatric interest*** was defined according to a double-level analysis. First, we investigated whether the disease or the medicinal product, for which the methodology was qualified and where specified in the QO, was addressed in a Pediatric Investigation Plan (PIP); in contrast, diseases or products included in a product waiver or the list of class waivers were considered without pediatric interest. PIPs and waivers were retrieved from the EMA website. Second, we assessed whether each methodology was already applied and used in pediatric studies by consulting clinicaltrials.gov^3^ and the literature. For QO concerning groups of methodologies, for example, groups of biomarkers qualified in a unique opinion for the same disease, we performed this check for each one.

The ***applicants*** were classified as either profit or non-profit. We also evaluated whether the development of the methodology was supported by any European public funding.

With regard to the ***type of methodology***, the classification was set based on the characteristics reported in the opinion letters, including the titles and keywords, given that the EMA does not provide any classification, conversely to the FDA.

The ***disease*** for which the methodology was referred was attributed to eight ***disease areas*** identified by EMA regulatory procedures, i.e., Pediatric Investigation Plans (PIPs), orphan designation, and European Public Assessment Report (EPAR), and then grouped according to the methodology detailed in our previous publications (21, 39). For methodologies applying to more than one area, we indicated “not applicable”. Additionally, we identified the methodologies addressing a specific disease.

We examined the list of issues released by the SAWP to identify any requests for further data from the SAWP on the use of the methodology in the pediatric population and the corresponding answers provided by the applicants. We also evaluated whether such requests led to the inclusion of pediatric data supporting such use. Moreover, we analyzed the comments from stakeholders and related EMA feedback released during the public consultation. The main purpose was to assess any changes in the final qualification opinion compared to the initial submitted draft, focusing on considerations related to the pediatric population, and to determine whether these changes were the result of comments provided by the stakeholders or whether they were influenced by the list of issues provided by the EMA.

The ***duration of the procedure*** was defined as the number of months from the date of adoption by the CHMP for release for public consultation to the date of adoption of the final opinion by the CHMP. We did not consider the time between the date of submission and the adoption by CHMP, as well as the date of the draft agreed by the SAWP and the adoption by CHMP, because some dates were missing, as detailed in the Supplementary material.

Data were collected and analyzed by four researchers; the final check was conducted by two researchers, who also discussed any possible disagreements to reach a consensus. Advice was requested from an expert in the pediatric research field (AC) in the case of methodologies applicable to a wide spectrum of diseases and from an expert in statistics (GR) in the case of statistical methodologies.

### Statistical analysis

We conducted a multi-level analysis to investigate data with a hierarchical structure and the effects of cluster-level variables and cluster-level variances. Generalized linear models (GLMs) were used for the analysis of time-series data. Differences were considered statistically significant when *p*-values were <0.05, while a *p* < 0.1 indicates weak evidence or a trend. SPSS statistical package version 29.0 (IBM Corp., Armonk, NY, United States) was employed for all statistical analyses.

## Results

### Pediatric interest in qualification procedures from 2008 to 2023

From the implementation of the EMA Qualification Procedure for Novel Methodologies in 2008 to December 2023, 27 applications received a positive opinion; one of them was a joint procedure with the FDA (EMEA/679719/2008). Three opinions published on the EMA website resulted in a “draft” (and therefore not considered for the analysis).

As detailed in the methods section, we analyzed the potential *pediatric interest* of all the 27 methodologies included in EMA qualification opinions. Most of the methodologies (19/27; 70%) were potential of interest to pediatric patients (see Table 1 for detailed information):

– 14 addressed a disease or a medicine included in a PIP;
– 1 not specifically intended for a medicinal product or disease was found to be used in pediatrics from the literature and clinicaltrials.gov; and
– four were considered of pediatric interest by experts.

**Table 1:** Qualification opinions with pediatric interest and their methodology type and disease area.

**ID qualification opinion**	**Methodology**	**Type**	**Therapeutic area**	**Included in a PIP**	**Pediatric data in QO**
EMADOC-1700519818-1127132	Stride velocity 95th centile as a primary endpoint in studies of ambulatory Duchenne muscular dystrophy studies	Endpoint	Neurology	Yes	Yes
EMADOC-1700519818-828910	Use of Enroll-HD (a Huntington's disease patient registry) as a data source and infrastructure support for post-authorization monitoring of medical products	Registry	Neurology	Yes	Yes
EMA/CHMP/SAWP/186420/2022	Islet autoantibodies (AAs) as enrichment biomarkers for type 1 diabetes (T1D) prevention clinical trials	Biomarker	Endocrinology	Yes	Yes
EMA/CHMP/SAWP/178058/2019	Stride velocity 95th centile as a secondary endpoint in Duchenne muscular dystrophy measured by a valid and suitable wearable device	Endpoint	Neurology	Yes	Yes
EMA/CHMP/SAWP/622564/2018	The European Cystic Fibrosis Society Patient Registry (ECFSPR) and CF pharmaco-epidemiology studies	Registry	Pulmonology	Yes	Yes
EMA/CHMP/SAWP/801872/2015	Pediatric ulcerative colitis activity index (PUCAI)	Endpoint	Gastroenterology	Yes	Yes
EMA/SA/00000104642	GFR slope as a validated surrogate endpoint for RCT in CKD	Endpoint	Nephrology	Yes	No
EMADOC-1700519818-946771	iBox Scoring System as a secondary efficacy endpoint in clinical trials investigating novel immunosuppressive medicines in kidney transplant patients	Endpoint	Infectious and immune system diseases	Yes	No
EMADOC-1700519818-907465	Prognostic Covariate Adjustment (PROCOVA™)	Statistical methodology	N/A	No	No
EMADOC-1700519818-808373	IMI PREFER	Research framework for patient preference studies	N/A	No	No
EMA/CHMP/SAWP/483349/2019	eSource Direct Data Capture (DDC)	Tool for data measurement/management	N/A	No	No
EMA/CHMP/SAWP/792574/2018	Cellular therapy module of the European Society for Blood and Marrow Transplantation (EBMT) Registry	Registry	Oncology	Yes	No
EMA/CHMP/SAWP/513571/2015	Ingestible sensor system for medication adherence as a biomarker for measuring patient adherence to medication in clinical trials	Biomarker	N/A	No	No
EMA/CHMP/SAWP/473433/2015	Total kidney volume (TKV) as a prognostic biomarker for use in clinical trials evaluating patients with autosomal dominant polycystic kidney disease (ADPKD)	Biomarker	Nephrology	Yes	No
EMEA/H/SAB/049/1/QO/2014/SME	*In vitro* hollow fiber system model of tuberculosis (HFS-TB)	Model for dose selection	Infectious and immune system diseases	Yes	No
EMA/CHMP/SAWP/757052/2013	MCP-Mod as an efficient statistical methodology for model-based design and analysis of phase II dose-finding studies under model uncertainty	Statistical methodology	N/A	No	No
EMA/CHMP/SAWP/120610/2020	Treatment effect measures when using recurrent event endpoints	Endpoint	Cardiology	Yes	No
EMA/CHMP/SAWP/283298/2010	ILSI/HESI submission of novel renal biomarkers for toxicity	Biomarker	Nephrology	Yes	No
EMEA/679719/2008 Rev. 1	Final conclusions on the pilot joint European Medicines Agency/Food and Drug Administration VXDS experience on qualification of nephrotoxicity biomarkers	Biomarker	Nephrology	Yes	No

The table details if the methodology addresses a disease or a medicine included in a PIP and the availability of pediatric data in the QO. Methodologies spanning more than one therapeutic area were indicated as N/A.

With regard to the eight methodologies without pediatric interest, seven of them referred to a disease included in a class or product waiver, and 1 was referred to a disease affecting children and included in PIPs, but the methodology was not applicable for children as it was not retrieved either in the literature or in clinicaltrials.gov.

### Type of applicant

**Figure 1:**
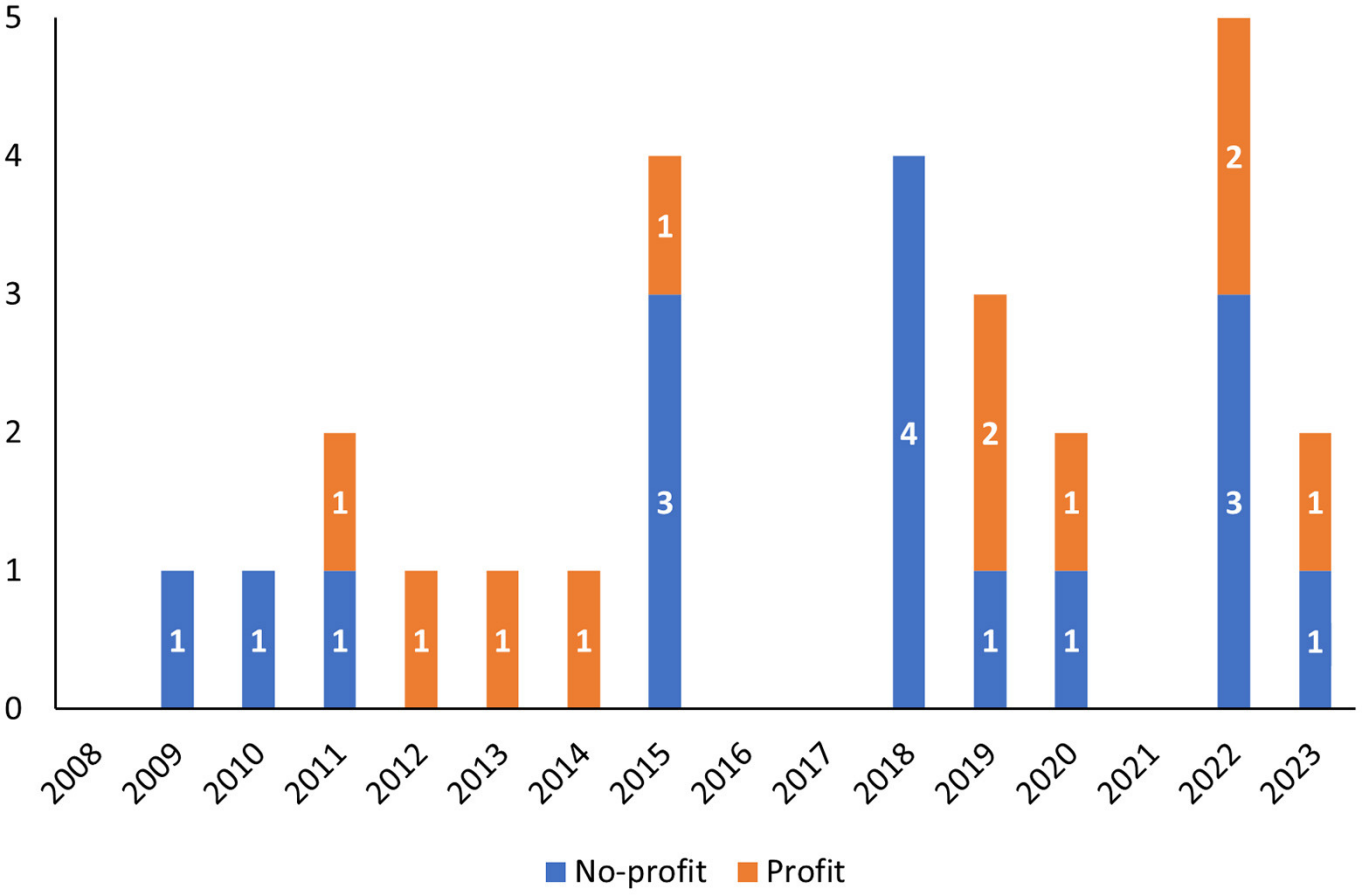
Qualification opinions from 2008 to 2023 grouped according to the year in which the opinion was adopted by CHMP and divided by the type of applicant (profit vs. non-profit).

No significant differences were found in terms of the type of applicants: 11 procedures were submitted by profit organizations, whereas 16 procedures were submitted by non-profit ones, with a quite regular alternation during the years (Figure 1). Similarly, QO procedures with pediatric interest were applied to both profit (8) and non-profit (11) applicants.

In addition, two opinion letters specifically mentioned that the methodology was fully or partly developed/studied in the context of European public funding, including the methodology “IMI PREFER” (EMADOC-1700519818-808373) in the context of the Innovative Medicine Initiative (IMI) project (grant agreement No. 115966) and the methodology “Proactive in COPD” (EMA/CHMP/SAWP/226829/2018) in the context of another IMI project (grant agreement No. IMI JU #115011).

### Types of methodology

**Figure 2:**
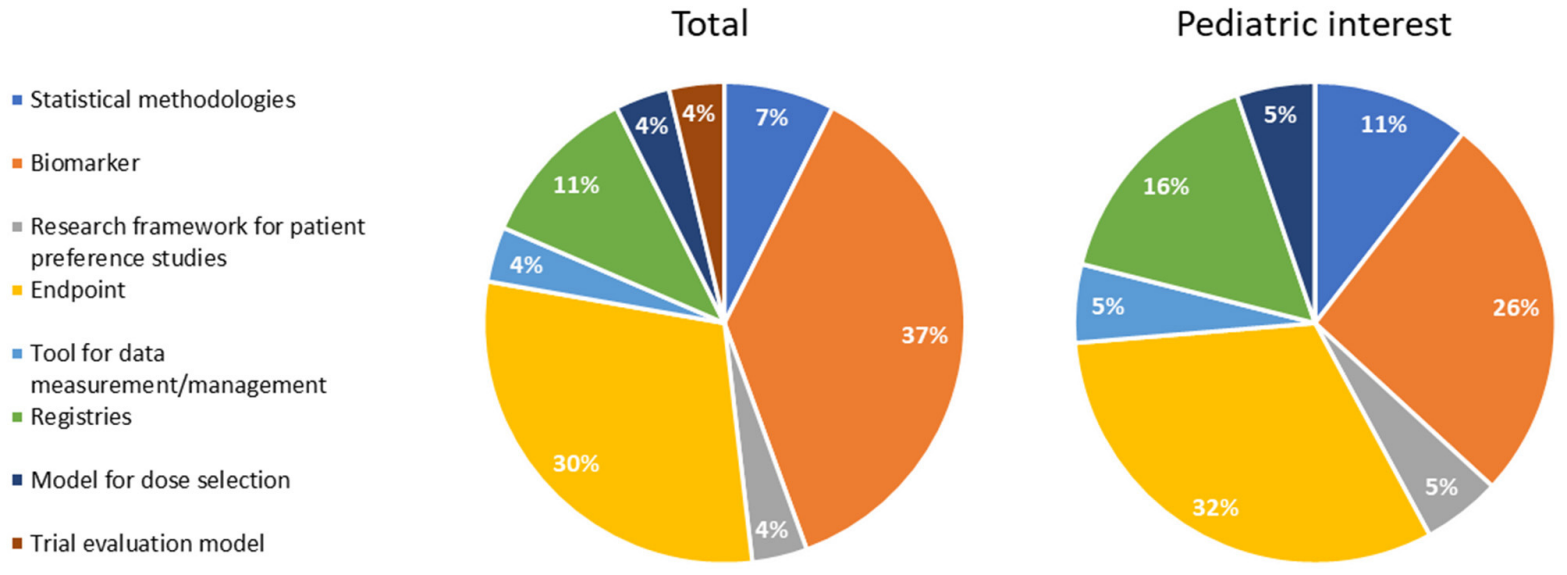
Qualification opinions grouped according to the type of methodology (total procedures and procedures of pediatric interest).

With regard to the types of methodologies, we classified them into the following categories: biomarker, endpoint, registry, statistical methodology, tool for data measurement/management, model (dose selection model, trial evaluation model), and research framework for patient preference study (Figure 2). Biomarkers, endpoints, and registries were the main types of innovative methodologies qualified by the EMA (37%, 30%, and 11%, respectively). The remaining qualified methodologies belonged to the other categories such as statistical methodology (7%), tool for data measurement/management (4%), research framework for patient preference studies (4%), dose selection (4%), and trial evaluation models (4%).

In line with the whole sample, endpoints, biomarkers, and registries were the main types of innovative methodologies with pediatric interest (32%, 26%, and 16%, respectively) (Figure 2).

### Diseases and disease areas addressed

**Figure 3:**
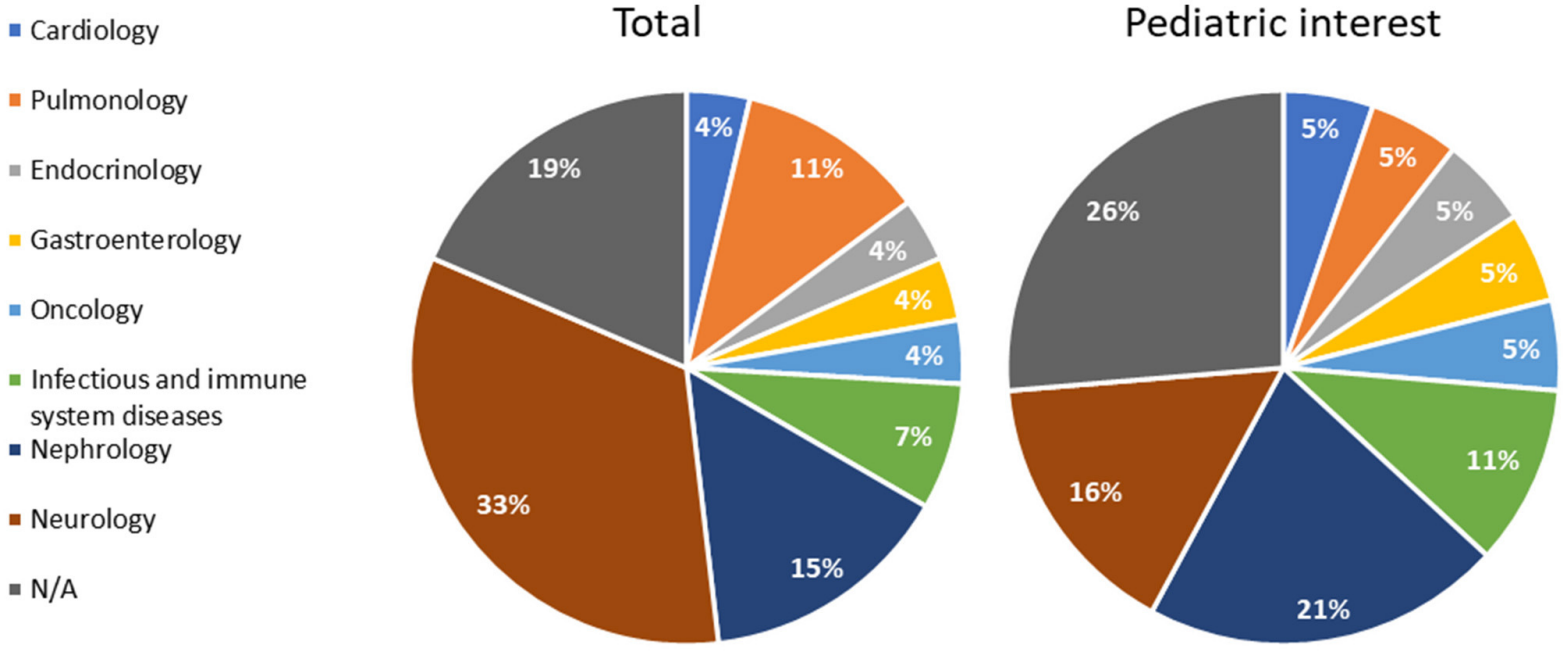
Qualification opinions grouped according to the disease area (total procedures and procedures of pediatric interest). N/A: methodologies applying to more than one area.

The qualified methodologies spanned eight different disease areas, namely, cardiology, endocrinology, gastroenterology, infectious and immune system disease, nephrology, neurology, oncology, and pulmonology, where neurology resulted in the most representation (33%), followed by nephrology (15%) and pulmonology (11%). Notably, 19% were not related to any specific area (Figure 3).

Out of 27 qualifications, 6 specifically addressed rare diseases: Duchenne Muscular Dystrophy, Huntington's disease, cystic fibrosis, and autosomal dominant polycystic kidney disease (ADPKD).

In line with the whole sample, in terms of disease areas, nephrology and neurology comprised the primary domains of methodologies with a pediatric focus, accounting for 21% and 16%, respectively (Figure 3). The five methodologies unrelated to a specific therapeutic area (26%) held potential pediatric interest (Figure 3, Table 1).

### Availability of pediatric data at the time of the opinion release

**Figure 4:**
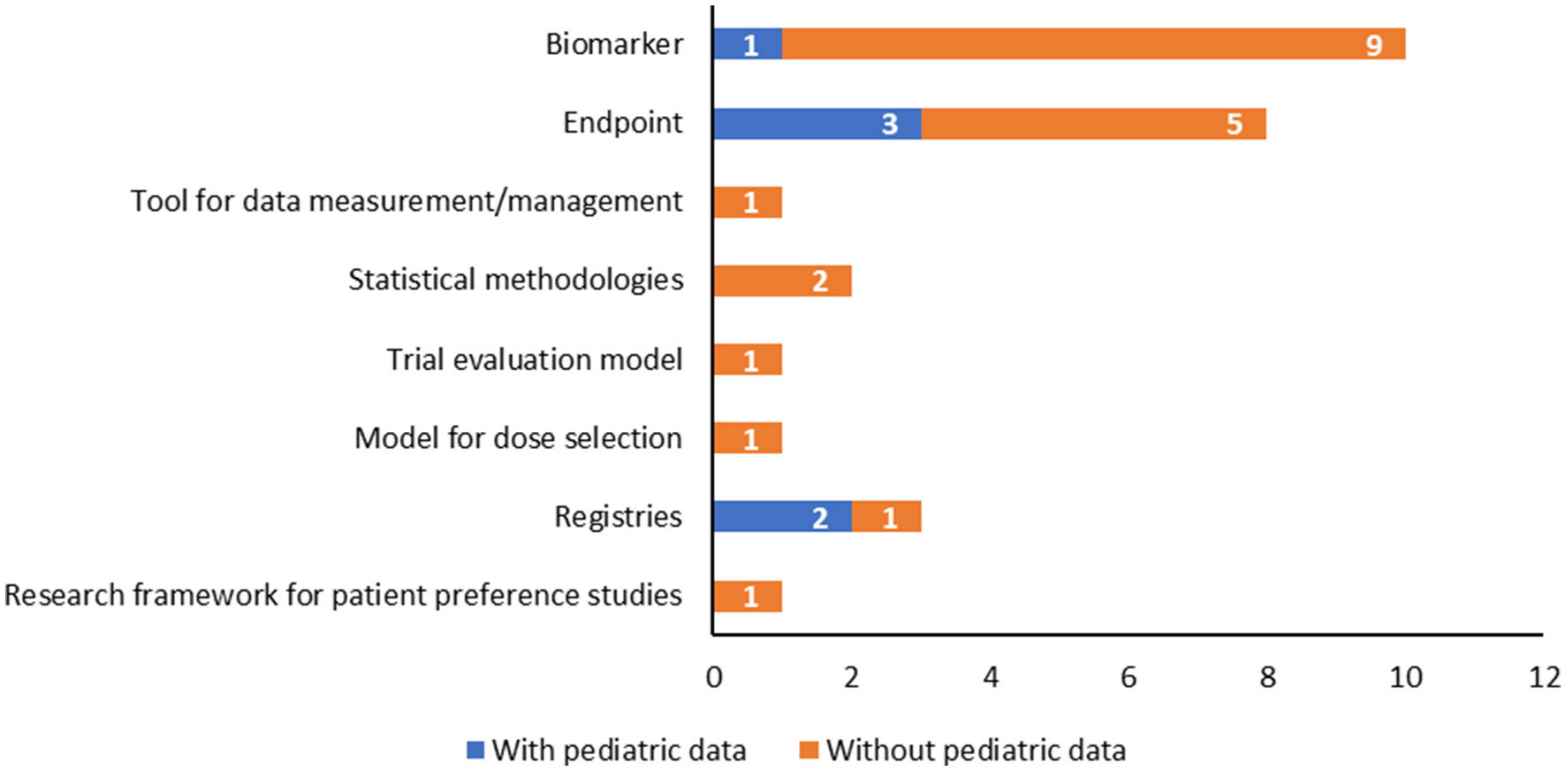
Qualification opinions with a pediatric interest grouped according to the type of methodology, highlighting those with pediatric data available.

Notably, only six of the total QOs reported pediatric data, as shown in Table 1. In particular, they are intended to assess both the safety and effectiveness of medicinal products and involve the use of patient registries (*n* = 2) or incorporate specific biomarkers (*n* = 2) and endpoints (*n* = 2) tailored to the pediatric population to reflect the disease's impact and progression (Figure 4, Table 1).

**Figure 5:**
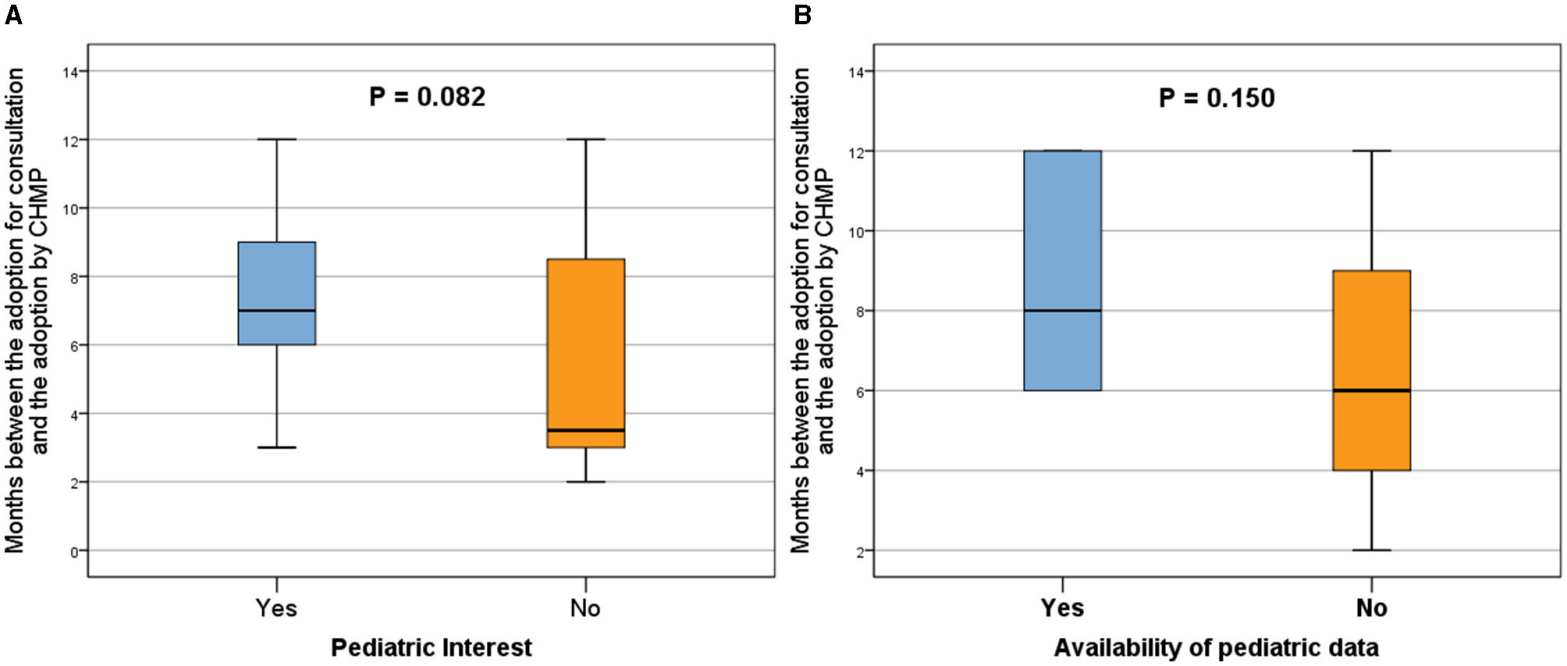
Analysis of the qualification procedure duration, defined as the months between adoption for consultation and adoption by CHMP, comparing the presence (Yes) or absence (No) of pediatric interest **(A)** and pediatric data in the QO **(B)**. Procedures with pediatric interest demonstrated an overall longer duration compared to those without pediatric interest **(A)**, and the inclusion of pediatric data extended the adoption period **(B)**. Box plots represent 25th, 50th, and 75th percentiles. Statistically significant differences were denoted by *p* < 0.05, while a *p* < 0.1 indicated a trend.

The examination of the list of issues released by the SAWP during the regulatory procedure, the applicants' corresponding answers, the stakeholders' comments, and the EMA's responses raised during the procedure highlighted that the only change between the draft and the final QO was related to the QO on an endpoint for Duchenne Muscular Dystrophy (DMD) studies (EMADOC-1700519818-1127132): in the list of issues, it was specifically required to provide updates from studies in the population below 5 years of age. During the consultation phase, the applicant submitted new data demonstrating that the performance of the tool was expected to be the same between 4- and 5-year-old children. Therefore, the age limit was lowered to 4 years of age in the adopted QO. This consultation phase and resulting modification did not result in a longer duration of the procedure (12 months overall, Figure 5).

For the other analyzed QOs, comments submitted by stakeholders or issues raised by the EMA related to pediatrics were duly acknowledged by the applicants; however, these comments did not lead to specific changes in the QOs (see Supplementary material for details).

### Duration of the procedure

The overall duration of the procedures with pediatric interest was longer than the overall duration of procedures without any pediatric interest (median time: 7.0 months vs. 3.5 months, respectively; *p* = 0.082; Figure 5A).

No significant differences were found in terms of the duration of the procedures between types of applicants, as detailed in the Supplementary material.

In parallel, the application procedures, including pediatric data, were adopted over a longer period (median time: 8.0 months vs. 6.0 months, respectively; *p* = 0.150; Figure 5B).

## Discussion

Over the past years, there has been a growing interest in employing innovative methodologies in biomedical research to gather evidence, as demonstrated by the literature (3, 40–42) and institutional public documents at the EU level (43). In addition, there has been an increased awareness of the need to adapt these methodologies for drug discovery and development and subsequent regulatory acceptance (1, 40). The current European Pharmaceutical Strategy recognizes the need for adapting scientific developments (genomics/personalized medicine) and technological transformation (data analytics and tools) to cutting-edge products, providing incentives for innovation, enhancing dialog among regulatory and other authorities, supporting collaborative projects on high-performance computing, artificial intelligence, and innovative trial designs, and a more patient-oriented medicine development (43).

In line with previous findings (40), our study shows that both profit and not-for-profit entities accessed the EMA qualification procedure. However, only a small percentage (about 10%) reached the full opinion. In fact, up to 2022, 209 requests for the qualification of novel methodologies were submitted to the EMA (20). In our analysis, only 27 applications received a positive opinion up to 2023.

Interestingly, biomarkers, endpoints, and registries emerged as the most represented methodologies qualified in the EU. Additionally, other types of methods were qualified as “regulatorily acceptable”, including statistical methodologies, tools for data measurement/management, *in vitro* pharmacokinetics models, disease progression models, and research frameworks for patient preference studies. These methodologies spanned across different therapeutic areas, where neurology is the most represented, with some specifically developed for rare diseases. This aspect highlights the relevance and applicability of these methodologies in addressing challenges associated with small populations, for example, rare diseases, underscoring their potential impact on advancing therapeutic interventions in these specialized areas.

Of note, only one procedure was jointly released with the FDA. As mentioned, a methodology can be assessed by the EMA and FDA together to issue its regulatory acceptance. The two agencies put in place different types of common/parallel submissions regarding the R&D of medicines for human use (PIP, ATMP, scientific advice, orphan designations, qualification procedures for biomarkers, and clinical outcome assessments).^4^ Interestingly, the FDA has a “qualification program” for drug development tools classified as animal models, biomarkers, clinical outcome assessments, and innovative science and technology approaches for new drugs. Conversely, the EMA does not provide any classification, making its procedure more “flexible” and allowing the inclusion of such research methodologies, such as registries. However, in line with previous findings (39), achieving harmonization between the two agencies still appears to be a lengthy process. The implementation of the ICH M15 guideline on model-informed drug development (44) would improve regulatory harmonization for model-based analyses as part of dossier submissions related to the development of pharmaceutical products.

If we look at pediatrics, our results demonstrate that a substantial proportion of novel, qualified methodologies hold significant promise for application in the pediatric population. Notably, also in the pediatric field, biomarkers, endpoints, and registries were the predominant types of innovative methodologies, underscoring their importance in advancing pediatric clinical research.

Remarkably, stride velocity 95th centile (SV95C; EMA/CHMP/SAWP/178058/2019) became the first digital endpoint regulatorily qualified in 2019 (45), and it is still the only one included in an EU qualification opinion. Digital biomarkers may capture patient-generated data and provide more objective measurements than traditional approaches, as they allow continuous and longitudinal data collection and the use of automated analysis for data interpretation. This aspect is particularly important for pediatric patients living with rare diseases, where therapeutic options are limited and need to be developed using a patient-focused approach to achieve the biggest impact. While digital technologies, including digital endpoints, are increasingly developed to support diagnosis, monitoring, or therapeutic interventions in clinical care, challenges arise in clinical validation due to the lack of specific guidelines. FDA guidance on patient-reported outcomes (46) could be adapted to ensure clinical validation when using digital tools in medical product development, particularly for pediatric patients with rare diseases, where patient-focused approaches are crucial.

However, our study also raises a critical concern: specific studies aimed at obtaining pediatric data are generally poor/lacking in qualification opinions. The observed discrepancy is concerning, despite the incentives and efforts implemented by the regulatory authorities in the EU to support pediatric R&D, such as the EU Pediatric Regulation (47). Only six of the examined methodologies were submitted for qualification with pediatric data. Moreover, our analyses showed that the inclusion of pediatric data in the procedure is associated with a longer duration of the overall process. However, the sample size was too small to detect a statistically significant difference.

The poor availability of data specifically generated from pediatric studies underscores the critical need for concerted efforts for the incorporation of pediatric data in research, emphasizing the importance of ensuring that innovative approaches are effectively translated into tangible benefits for pediatric patients.

Another missed opportunity for the inclusion of children in clinical research is represented by the IMI PREFER case (EMADOC-1700519818-808373). The PREFER (Patient Preferences in Benefit-Risk Assessments during the Drug Life Cycle) framework primarily focuses on incorporating patient preferences in benefit-risk assessments for medical treatments. While the framework highlights the importance of patient involvement, including preferences from various patient populations, based on our latest knowledge, it does not specifically focus on children. Furthermore, it is worth noting that the MSCOA (Multiple Sclerosis Clinical Outcome Assessment), as referenced in QO EMA/CHMP/SAWP/74371/2020, has been designed to capture clinical outcome assessments in patients with multiple sclerosis. However, it was not expressly tailored for children, despite its potential relevance for the pediatric population. Obtaining pediatric data would allow for an understanding of the efficacy and safety of treatments for children affected by multiple sclerosis.

Further interest in the pediatric field might come from the fact that some chronic diseases affecting adults have rare genetic forms with a pediatric onset, as in the case of chronic heart failure in children affected by congenital heart defects or cardiomyopathies (48). In these circumstances, even if the disease does not have a pediatric interest *per se*, early identification and intervention in pediatric patients can significantly impact their long-term outcomes. This emphasizes, on the one hand, the interconnected nature of pediatric and adult medicine in addressing complex chronic diseases and, on the other hand, the importance of a comprehensive approach to medical research and practice that considers the entire spectrum of human life, from infancy to adulthood.

The raised concern is pervasive across various domains of pediatric research, highlighting the imperative to allow the implementation of the continuous advancement of science and innovation in pediatric research. This objective could be achieved, as mentioned above, through the adoption of optimization of clinical study designs, innovative statistical approaches, extrapolation, and other pharmacometric approaches across pediatric ages to support their use in pediatrics (23, 25, 31, 49). Currently, it is well known that the use of pharmacometric approaches can streamline R&D while maintaining the reliability of data. This aspect would also be applicable to the need to include pediatric data without relying solely on the generation of new data. For example, extrapolation methodologies could be used to infer pharmacokinetics, pharmacodynamics, and efficacy from a reference patient population or from animals, another compound or disease (50). The application of these strategies would maximize the usefulness of existing knowledge with the minimum number of subjects enrolled, thus making it more comprehensive and worthwhile to include pediatric data in the qualification procedure.

Additionally, innovative methods for obtaining informed consent and assent or their updates (e.g., digital consent and assent) could be adopted to improve pediatric research. Similarly, approaches for collecting blood samples or other types of biological material could be updated, potentially minimizing pain, discomfort, and distress in pediatric studies (38).

Further exploration of ways to strengthen the research framework in the pediatric field is essential to ensure the highest standards of care and safety for pediatric participants.

At the EMA level, several initiatives are in place to support the application of new and innovative methods in the research of medicines, especially in areas concerning small populations, such as rare diseases and pediatric subjects. EMA working parties collaborate with scientific committees to assist companies and researchers in this effort. For example, the EMA has established the Innovation Task Force (ITF) (51) to ensure coordination across the agency and to serve as a platform for early dialog with applicants regarding innovative aspects in the development of medicines. Crucial insights and guidance may derive from the actions and initiatives led by this task force and the above-mentioned pharmaceutical strategy for Europe (43), which actively support the integration of innovative methods in clinical trials and, more broadly, in the overall development of medicines. Further expectations come from the Accelerating Clinical Trials in the EU (ACT EU)^5^ initiative. It has been set up in the EU to develop a competitive center for innovative clinical research. Therefore, ACT EU does represent an opportunity to bring innovation to clinical research, particularly in multi-center trials. Pediatric networks, such as c4c (conect4children, a large collaborative European network aimed at facilitating the development of new drugs and other therapies for the entire pediatric population),^6^ TEDDY (the European Network of Excellence for Pediatric Research),^7^ specialistic pediatric networks, and the other members of the European Network of Pediatric Research at the European Medicines Agency (Enpr-EMA),^8^ as well as EPTRI,^9^ the European Pediatric Translational Research Infrastructure, and the other pan-European Research Infrastructures, ECRIN,^10^ BBMRI,^11^ and EATRIS,^12^ could contribute providing and updating specific tools and services to conduct pediatric studies (38).

Even more recently, the European Commission has funded two new projects under the call “Modeling and simulation to address regulatory needs in the development of orphan and pediatric medicines” (HORIZON-HLTH-2023-IND-06-04). These projects fully addressed the regulatory acceptance of innovative research methodologies in pediatric research. Their outputs could then provide meaningful insights into the relevant field.

Another way to move forward could be to strengthen the awareness and coordination between EU regulatory procedures, for example, orphan designation, PIP, and clinical trial applications. In all these regulatory submissions, specific references could be made if a “qualified” innovative methodology has been used. Such a regulatory provision could improve awareness of the regulatory acceptance of a “research method” not only among researchers, medicine developers, and other applicants but also among regulators. In addition, to ensure that pediatrics is not left behind when innovative methodologies are developed, an explicit statement on the presence or absence of pediatric data could be included in the application form when defining the context of the use of the methodology. This suggested approach would better delineate the usefulness and applicability of the methodology in the pediatric field. Very recently, a checklist to guide the structure and content of qualification applications and a periodical re-evaluation of the qualified elements to ensure the standards that are maintained over time has been proposed.^13^ If applied, such modifications would represent an occasion to implement pediatric-specific information in the procedure.

Overall, our results support the importance of implementing innovative methodologies into regulatory-compliant pediatric research activities. In this context, dedicated pediatric research infrastructures could assist in addressing the data gaps in pediatric research, offering regulatory support and strategic advice throughout the research process. These infrastructures play a crucial role in designing *ad hoc* pediatric methodologies or extending and validating existing ones for pediatrics.

## Data availability statement

The raw data supporting the conclusions of this article will be made available by the authors, without undue reservation.

## Author contributions

VG: Data curation, Methodology, Writing – original draft. AB: Data curation, Formal analysis, Investigation, Methodology, Visualization, Writing – review & editing. ST: Data curation, Formal analysis, Investigation, Writing – original draft, Writing – review & editing. GR: Formal analysis, Writing – review & editing. ET: Data curation, Investigation, Writing – review & editing. DB: Supervision, Writing – review & editing. AC: Conceptualization, Supervision, Writing – review & editing.

## Footnotes

^1^ https://www.ema.europa.eu/en/partners-networks/international-activities/cluster-activities

^2^ https://www.ema.europa.eu/en

^3^ https://clinicaltrials.gov/

^4^ https://www.orpha.net/consor/cgi-bin/index.php

^5^ https://www.ema.europa.eu/en/human-regulatory-overview/research-and-development/clinical-trials-human-medicines/accelerating-clinical-trials-eu-act-eu

^6^ https://conect4children.org/

^7^ https://www.teddynetwork.net/

^8^ https://www.ema.europa.eu/en/partners-networks/networks/european-network-paediatric-research-european-medicines-agency-enpr-ema

^9^ https://eptri.eu/

^10^ https://ecrin.org/

^11^ https://www.bbmri.it/

^12^ https://eatris.eu/

^13^ https://www.ema.europa.eu/en/events/joint-heads-medicines-agencies-hma-european-medicines-agency-ema-multistakeholder-workshop-patient-registries
