# Supplementary material for: Innovative research methodologies in the EU regulatory framework: an analysis of EMA qualification procedures from a pediatric perspective
Source: Front Med (Lausanne). 2024 Mar 28;11:1369547. doi: 10.3389/fmed.2024.1369547 (PMC11007141; doi:10.3389/fmed.2024.1369547)
Supplement: Supplementary file 1 [file Data_Sheet_1.PDF]

Supplementary Material

1 Statistical analysis on the duration of the Qualification Opinion procedure of the whole sample (N = 27)

Supplementary Table 1. Duration of the Qualification Opinion (QO) procedures: time between the adoption for consultation and the adoption by CHMP.

Descriptives		Months between the adoption for consultation and CHMP adoption	
		Statistic	Std. Error
Mean		7.11	.712
95% Confidence Interval for Mean	Lower Bound	5.65	-
	Upper Bound	8.57	-
5% Trimmed Mean		6.86	-
Median		6.00	-
Variance		13.641	-
Std. Deviation		3.693	-
Minimum		2	-
Maximum		18	-
Range		16	-
Interquartile Range		5	-
Skewness		.988	.448
Kurtosis		1.333	.872

2 Type of Applicant Analysis of the whole sample (N = 27)

Supplementary Table 2. Descriptives on the duration of the QO procedure according to the type of applicant (profit and no-profit).

Type of Applicant		Months between the date of adoption for consultation and the adoption by CHMP
Profit	N	11
	Mean	6.91
	Std. Deviation	3.300
	Median	7.0
	Minimum	3
	Maximum	12
No-profit	N	16
	Mean	7.25
	Std. Deviation	4.041
	Median	6.0
	Minimum	2
	Maximum	18
Independent Samples t-test p-value		0.819
Wilcoxon test p-value		0.980

Supplementary Figure 1. Time between the adoption for consultation and the adoption by CHMP.

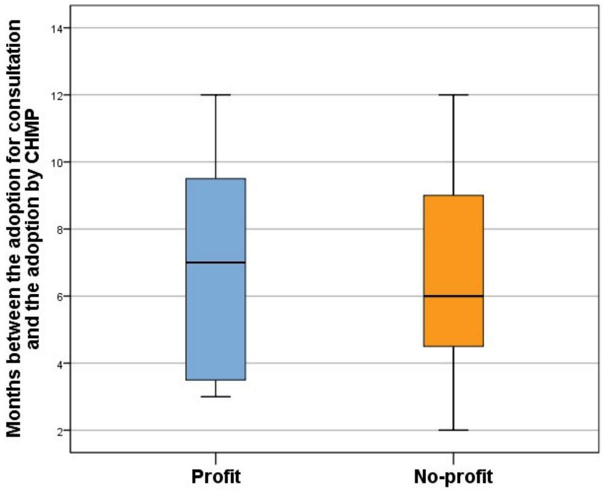

3 Potential pediatric interest Analysis of the whole sample (N = 27).

Supplementary Table 3. Duration of the QO procedure with and without interest for pediatrics.

Potential pediatric interest		Months between the adoption for consultation and CHMP adoption
YES	N	19
	Mean	7.47
	Std. Deviation	2.568
	Median	7.0
	Minimum	3
	Maximum	12
NO	N	8
	Mean	6.25
	Std. Deviation	5.701
	Median	3.5
	Minimum	2
	Maximum	18
Independent Samples t-test p-value		0.443
Wilcoxon test p-value		**0.082**

4 Availability of Pediatric Data Analysis of the whole sample (N = 27).

Supplementary Table 4. Duration of the QO procedure with and without pediatric data.

Availability of Pediatric Data		Months between the adoption for consultation and CHMP adoption
YES	N	6
	Mean	8.67
	Std. Deviation	3.011
	Median	8.0
	Minimum	6
	Maximum	21
NO	N	21
	Mean	6.67
	Std. Deviation	3.812
	Median	6.0
	Minimum	2
	Maximum	18
Independent Samples t-test p-value		0.250
Wilcoxon test p-value		0.150

5 Public consultations related to the Qualification Opinions with pediatric data.

Supplementary Table 5. List of issues and comments received during the public consultations and EMA replies related to the Qualification Opinions with pediatric data.

TOOL	QO DRAFT	LIST OF ISSUES	COMMENTS RECEIVED DURING PUBLIC CONSULTATION	EMA REPLY TO COMMENTS RECEIVED DURING PUBLIC CONSULTATION	ADOPTED QO
Stride velocity 95th centile as primary endpoint in studies in ambulatory Duchenne Muscular Dystrophy studies	c. Quantitative evidence: Data from the 45 European DMD patients as assessed in the previous qualification request are supplemented with data from 80 additional patients from US, EU and Australia (n=125 patients 2597 overall) either stable or starting on corticosteroids. Data from 66 healthy age-matched controls are also presented. The data originate	Question #11 Please provide an update on studies that could add data for qualification and if these could allow an assessment in the population below 5 years of age and a more robust derivation of an anchor based MCT.			c. Quantitative evidence: Data from the 45 European DMD patients as assessed in the previous qualification request are supplemented with data from 80 additional patients from US, EU and Australia (n=125 patients overall) either stable or starting on corticosteroids. Data from 66 healthy age-matched controls are also presented. The data originate from 7 clinical trials including Natural History Data and data from in-clinic patients (Appendix 7.8). In the initial

TOOL	QO DRAFT	LIST OF ISSUES	COMMENTS RECEIVED DURING PUBLIC CONSULTATION	EMA REPLY TO COMMENTS RECEIVED DURING PUBLIC CONSULTATION	ADOPTED QO
	from 7 clinical trials including Natural History Data and data from in clinic patients (Appendix 7.8). As only data for patients older than 5 years are provided, no conclusions on suitability for a younger age group can be drawn				submission only data for patients of 5 years and older were provided. This was the reason to limit the context of use to children ≥5 years of age. During the consultation phase new data were submitted, indicating that compliance is not different for DMD subjects between 4 and 5 years of age (n=29) as compared to DMD subjects between 5 and7 years of age (n=100). It is not expected that for children between 4 and5 years of age, who are able to wear the device appropriately, the performance of the SV95C is different. Hence, there is no

TOOL	QO DRAFT	LIST OF ISSUES	COMMENTS RECEIVED DURING PUBLIC CONSULTATION	EMA REPLY TO COMMENTS RECEIVED DURING PUBLIC CONSULTATION	ADOPTED QO
					objection to the lower age limit to 4 years of age.
Use of Enroll-HD (a Huntington's disease patient registry) as a data source and infrastructure support for post-authorisation monitoring of medical products					no changes between the draft and the adopted QO

TOOL	QO DRAFT	LIST OF ISSUES	COMMENTS RECEIVED DURING PUBLIC CONSULTATION	EMA REPLY TO COMMENTS RECEIVED DURING PUBLIC CONSULTATION	ADOPTED QO
Islet Autoantibodies (AAs) as Enrichment Biomarkers for Type 1 Diabetes (T1D) Prevention Clinical Trials			Whilst the current analysis assesses high-risk HLA (present/absent) and the presence of two or more antibodies as covariates, it does not distinguish between the kinds of antibodies or HLA DR3/DR4. It might be worthwhile to point out that the risk of developing T1D within 5 years for an 8-year-old child with two or more islet antibodies is expected to differ depending on the type of antibodies and which antibody occurred first. For instance, the presence of ZnT8A is associated with	The derived baseline utilized in the modeling analysis by definition includes subjects positive for any two or more islet autoantibodies at the time of risk assessment. Given the context of use, it is not critical to characterize the time history of seroconversion. The subtypes included in the modeling analysis were limited to those available in the underpinning datasets. As such, during the baseline covariate analysis, HLA status was included as binary presence or absence of the included HLA types. High risk HLA subtype did not show a significant effect on overall survival and was subsequently dropped for further analyses.	no changes between the draft and the adopted QO

TOOL	QO DRAFT	LIST OF ISSUES	COMMENTS RECEIVED DURING PUBLIC CONSULTATION	EMA REPLY TO COMMENTS RECEIVED DURING PUBLIC CONSULTATION	ADOPTED QO
			older age at diagnosis (Salonen et al. 2013).		
Stride velocity 95th centile as a secondary endpoint in Duchenne Muscular Dystrophy measured by a valid and suitable			Activity monitors are proposed to give a continuous, long term recording of the child's function, based around normal activity in the home environment. It is interesting that the proposed endpoint,	Overall no changes required.	no changes between the draft and the adopted QO

TOOL	QO DRAFT	LIST OF ISSUES	COMMENTS RECEIVED DURING PUBLIC CONSULTATION	EMA REPLY TO COMMENTS RECEIVED DURING PUBLIC CONSULTATION	ADOPTED QO
wearable device			found to be most useful by the applicant is gait velocity, rather than a more global measure of steps taken or distance walked over the recording period.		
Stride velocity 95th centile as a secondary endpoint in Duchenne Muscular Dystrophy measured by a valid and suitable wearable device	The paediatric word was absent in the draft		For the paediatric neurologist comments, please can you propose a definition for an individual 'loss of ambulation' based on the actimyo collected data, e.g. < 20% of total baseline distance walked, or other measures.		The paediatric word was added in the adopted QO
	The paediatric word was absent in the draft		For the paediatric neurologist comments, please can you provide the data tables and		The paediatric word was added in the adopted QO

TOOL	QO DRAFT	LIST OF ISSUES	COMMENTS RECEIVED DURING PUBLIC CONSULTATION	EMA REPLY TO COMMENTS RECEIVED DURING PUBLIC CONSULTATION	ADOPTED QO
			corresponding correlational analysis in the DMD patients.		
	The paediatric word was absent in the draft		For the paediatric neurologist comments; do you have more longitudinal data accrued, analysed and available at this stage for tables 6,7,and 8? Whilst these are being collected for the primary endpoint, availability of these data now would further strengthen the current qualification opinion		The paediatric word was added in the adopted QO.

TOOL	QO DRAFT	LIST OF ISSUES	COMMENTS RECEIVED DURING PUBLIC CONSULTATION	EMA REPLY TO COMMENTS RECEIVED DURING PUBLIC CONSULTATION	ADOPTED QO
The European Cystic Fibrosis Society Patient Registry (ECFSPR) and CF Pharmaco-epidemiology Studies			It is mentioned that there are pediatric patients included in this registry, and we'd like to ask for further clarification:	Acknowledged.<br>Please note that the opinion already makes reference to the broad coverage of the CF population (lines 334-345), the widely established use of newborn screening in the EU (339-341) and highlights that these 'real world' populations are followed/assessed over extended periods of time (529-530).	no changes between the draft and the adopted QO.
Paediatric ulcerative colitis activity index (PUCAI)			Additional points that would be helpful to clarify are discussed as follows:<br>The description of the potential conditions for extrapolation of the effect on mucosal healing to paediatric clinical trials and thereby waiving endoscopy	a) Extrapolation is a completely distinct approach in paediatric IBD, which is not related to the qualification of an outcome measure, which itself implies the conduct of studies. Therefore, any statements on extrapolation are not needed. Further guidance for extrapolation will be dealt with in the general guidance planned to be published for	no changes between the draft and the adopted QO.

TOOL	QO DRAFT	LIST OF ISSUES	COMMENTS RECEIVED DURING PUBLIC CONSULTATION	EMA REPLY TO COMMENTS RECEIVED DURING PUBLIC CONSULTATION	ADOPTED QO
			is particularly vague. Acknowledging the statement that this topic is outside the scope of this qualification opinion, is EMA planning to provide further guidance in this subject?	extrapolation, and in the ongoing revision of the IBD guidelines (EMA/129698/2012 and EMA/CHMP/327812/2014)	
